# Recent Treatment Advances and New Trials in Adult Nephrotic Syndrome

**DOI:** 10.1155/2017/7689254

**Published:** 2017-05-03

**Authors:** Eva Königshausen, Lorenz Sellin

**Affiliations:** Department of Nephrology, University Hospital, Medical Faculty, Heinrich-Heine University, Moorenstrasse 5, 40225 Duesseldorf, Germany

## Abstract

The etiology of nephrotic syndrome is complex and ranges from primary glomerulonephritis to secondary forms. Patients with nephrotic syndrome often need immunosuppressive treatment with its side effects and may progress to end stage renal disease. This review focuses on recent advances in the treatment of primary causes of nephrotic syndrome (idiopathic membranous nephropathy (iMN), minimal change disease (MCD), and focal segmental glomerulosclerosis (FSGS)) since the publication of the KDIGO guidelines in 2012. Current treatment recommendations are mostly based on randomized controlled trials (RCTs) in children, small RCTs, or case series in adults. Recently, only a few new RCTs have been published, such as the Gemritux trial evaluating rituximab treatment versus supportive antiproteinuric and antihypertensive therapy in iMN. Many RCTs are ongoing for iMN, MCD, and FSGS that will provide further information on the effectiveness of different treatment options for the causative disease. In addition to reviewing recent clinical studies, we provide insight into potential new targets for the treatment of nephrotic syndrome from recent basic science publications.

## 1. Introduction

Nephrotic syndrome is characterized by gross proteinuria, hypoalbuminemia, hyperlipidemia, and peripheral edema [1]. The etiology of nephrotic syndrome in adults is complex and ranges from primary glomerulonephritis to secondary forms [1]. This review will focus on new therapeutic advances in treating primary forms of nephrotic syndrome in adult patients. Primary forms of nephrotic syndrome in adults are comprised of three histological disease entities: idiopathic membranous nephropathy (iMN), minimal change disease (MCD), and focal segmental glomerulosclerosis (FSGS).

Primary nephrotic syndrome often affects younger patients. In this young population, primary glomerulopathies are the most frequent cause of end stage renal disease (ESRD) [2]. The incidence of primary forms of nephrotic syndromes in adults however is low (0.6–1.2 cases/100.000 adults depending on the underlying histological disease) [2]. Hence, little is known about the therapeutic strategies that can impact this adult population. Therefore, the identification of novel therapeutic strategies for nephrotic syndrome in adults is essential to prevent or delay ESRD.

The basis for therapy of primary nephrotic syndrome is mostly of supportive nature. Supportive strategies include antihypertensive and antiproteinuric therapy and dietary recommendations [3]. Patients with nephrotic syndrome are also at increased risk to develop thromboembolism. In patients with membranous nephropathy, the adjusted hazard ratio for thromboembolism was 10.8 compared to patients with IgA nephropathy [4]. In contrast, for patients with FSGS the hazard ratio was 5.9 [4]. Hence, anticoagulant therapy is recommended in patients with a primary nephrotic syndrome, especially in iMN and serum albumin < 2,5 mg/dl [1]. In 2014, Lee et al. proposed a practical approach to prophylactic anticoagulation therapy in patients with iMN [5]. The presented model takes into account the serum albumin concentration, the individual patient's bleeding risk, and the risk tolerance as reflected by the selected benefit-to risk ratio (http://www.gntools.com) [5].

We will focus on major therapeutic advances in iMN, MCD, and FSGS as causes for primary nephrotic syndrome in adults. Due to space limitations, we had to focus on selected references in this review.

## 2. Major Therapeutic Advances

### 2.1. Idiopathic Membranous Nephropathy (iMN)

IMN was recently identified as an autoimmune disease against the M-type phospholipase A2 receptor (PLA2R) in 70–80% of the white population [6]. As for most autoimmune diseases, genetic polymorphisms predispose to the disease. Polymorphisms of HLA-DQA1 were associated with polymorphisms in the PLA2R in a genome wide association study (GWAS) [7]. Even more recently, thrombospondin type-1 domain-containing 7A (THSD7A) was established as a second although less common autoantigen in iMN [8]. The fact that 10–20% of patients are seronegative for PLA2R and THSD7A indicates that there are probably more yet unknown autoantigens. Spontaneous remission of iMN is observed in up to 30% of the patients within 14 months [9]. Indications for immunosuppressive therapy for iMN therefore depend on the level of proteinuria (>4 g/24 h), rate of GFR decline, and complications of the nephrotic syndrome [1]. Drop of PLA2R antibody titers usually precede reduction in proteinuria [2, 10, 11]. Recently, De Vriese et al. proposed a serology based approach to diagnosis, prognosis, and treatment monitoring of patients with iMN [12]. This review takes into account that the traditional proteinuria based approach to treatment decisions potentially lags months behind a change in immunological activity and that otherwise proteinuria may reflect an irreversible damage to the glomerular filter without active disease [12]. As changes in PLA2R antibody titers (and potentially THSD7A antibody titers) tightly correlate with disease activity, this approach may help to increase diagnostic and prognostic accuracy and reduce unnecessary immunosuppressive therapy [12]. However, the serology based approach needs to be validated in a randomized controlled trial (RCT) compared to the traditional approach before replacing it in clinical practice.

#### 2.1.1. Guideline Recommendations for Therapy of iMN

If the criteria for immunosuppressive therapy are met, KDIGO guidelines recommend to primarily use alkylating agents (cyclophosphamide) in combination with corticosteroids for 6 months [1]. Alternatively, calcineurin inhibitors are also recommended in addition to corticosteroids for 6 months [1].

#### 2.1.2. Clinical Advances and Randomized Controlled Trials (RCT) in iMN

IMN shows a significant amount of spontaneous remission (see above). A Dutch RCT investigated 26 patients with nephrotic syndrome who had a normal glomerular filtration rate (GFR). One group received early immunosuppression (cyclophosphamide) while the other group was followed until their serum creatinine values increased by at least 25% [13]. They reported that there was no difference in remission rates, the course of GFR loss, or complications after 72 ± 22 months. A retrospective analysis of 254 patients with iMN revealed that after ten years seven patients (3%) developed ESRD, 25 (10%) died, 52 (20%) achieved full remission, and 90 (35%) achieved partial remission if immunosuppression was given only if GFR declined or serious complications of the nephrotic syndrome occurred [14]. These studies therefore support more limited application of immunosuppressive treatment in patients with iMN and normal GFR.

In patients with iMN and reduced GFR at diagnosis, chlorambucil and prednisolone were superior in delaying GFR loss compared to treatment with cyclosporine or supportive measures alone [15]. However, patients treated with chlorambucil and prednisolone experienced a significantly higher rate of adverse events (AEs) [15]. Recent data indicates that the cancer incidence in patients treated with alkylating agents triples compared to that in the nonexposed population [16]. In addition, a systematic review and meta-analysis of 36 trials concluded that alkylating agents in combination with corticosteroids protect from GFR loss [17]. In addition, cyclosporine and mycophenolate mofetil were not superior to alkylating agents (mostly cyclophosphamide) and prednisolone [17].

In search for new therapeutic approaches to treat iMN and other autoimmune diseases, researchers have become interested in the potential of the adrenocorticotropic hormone (ACTH) because only minor side effects were reported in an earlier study [18]. A more recent prospective, open label cohort study with synthetic ACTH (Synacthen®) compared 20 patients with iMN and high risk for progression with a historical control group treated with cyclophosphamide and prednisolone [19]. This study found that synthetic ACTH is less effective in inducing remission in high-risk patients with iMN compared to cyclophosphamide (cumulative remission in ACTH 55% and cyclophosphamide plus corticosteroids 95%) [19]. In addition, AEs in the ACTH cohort were very high (95%, 25% needed hospitalization). Therefore, the authors advised against treatment with ACTH in iMN. In contrast, a dose escalation study using natural ACTH gel (HP Acthar® gel) found a significant reduction in proteinuria with an acceptable AE profile [20]. A RCT testing ACTH versus placebo in patients with iMN at high risk of progression is in progress (clinicaltrials.gov identifier: NCT01386554) and will clarify the role of ACTH in the treatment of iMN in this patient group.

Recent pathophysiological advances in understanding the role of autoimmunity in iMN have resulted in new therapeutic studies targeting B-cells in iMN. Observational studies in the past had indicated that rituximab (375 mg/m^2^ weekly for 4 weeks or 1 g every 14 days ×2 or 1 g single dose) was effective as conventional treatment with reduced AEs in short observational periods [21–23]. However, a dose of 375 mg/m^2^ only once or twice showed a poor outcome in inducing remission (<50%) with preserved renal function (GFR > 60 ml/min) [24]. So far only one published RCT (Gemritux) has investigated conservative therapy (nonimmunosuppressive antiproteinuric therapy) in comparison with rituximab (375 mg/m^2^ at days 1 and 8 in patients with nephrotic proteinuria, normal range creatinine) [25]. In the Gemritux trial, the rituximab cohort showed a remission rate of 35% versus 21% in the control group after 6 months (*p* = nonsignificant). In the follow-up period, the remission rates increased to 65% (rituximab) and control (34%) with a significant difference favouring the rituximab group while AEs were comparable. Further RCTs investigating the role of rituximab in the treatment of iMN are ongoing, for example, the MENTOR trial (persistent proteinuria > 5 g/day comparing cyclosporine monotherapy for 12 months with rituximab 1 g on days 1 and 15 repeated at 6 months (clinicaltrials.gov NCT01180036)) [26]. Furthermore, the STARMEN trial is comparing corticosteroids and cyclophosphamide for 6 months with 6 months of tacrolimus followed by a single dose of rituximab (1 g at days 1 and 15, repeated at 6 months independent of CD19 count) (clinicaltrials.gov NCT01180036) [27]. A pilot study using a combination of rituximab and cyclosporine, with the latter tapered off after 6 months while giving repeated rituximab infusion at that point, is also ongoing (clinicaltrials.gov NCT00977977).

#### 2.1.3. Potential New Targets and Therapeutic Strategies in iMN

Unfortunately, current treatment options for iMN have a decent percent of nonresponsive patients and posttreatment relapse rates of 15–30% [1]. To reduce relapse rates, Cattran and Brenchley suggest investigating maintenance therapy with azathioprine or mycophenolate mofetil after induction therapy with alkylating agents or rituximab, comparable to ANCA-associated vasculitis [28]. An ongoing trial is examining the effectiveness of Chinese herbal medicine (Quing-ReMoShen granules) in combination with RAAS-blockers on the reduction of proteinuria and T-cell function in iMN (clinicaltrials.gov NCT01845688). Epitope mapping of the PLA2R and THSD7A autoantibodies will potentially help to develop immunoabsorption columns to deplete autoantibodies [28]. In a retrospective case series, plasma exchange (4 PE against albumin) in combination with intravenous immunoglobulins (20 g) and rituximab (375 mg/m^2^) was able to induce partial remission in 10 high-risk patients (more than 3 relapses, proteinuria > 3.5 g/g creatinine, and anti-PLA2R antibody titer > 1 : 500 besides treatment with immunosuppressive therapy for more than 12 months) [29].

Furthermore, a role for T-cells in iMN has been proposed [30]. In patients treated with rituximab, regulatory T-cells (Tregs) increased up to 10-fold, an increase that persisted in responders after 12 months [31]. Therefore, modulation of T-cell function with, for example, soluble T-cell peptides to tolerize T-cells and/or enhance Tregs to downregulate the anti-PLA2R/THSD7A response is promising a new treatment strategy for iMN [28].

### 2.2. Minimal Change Disease

Minimal change disease (MCD) accounts for 10–15% of patients with idiopathic nephrotic syndrome in adults [32]. Pathologically, it is characterized by minimal changes on light microscopy and foot process effacement on electron microscopy [1]. With regard to the pathogenesis of the disease, it is still debated whether MCD and focal segmental glomerulosclerosis (FSGS) are separate disease types or a disease continuum [2, 32]. For both, circulating factors have been proposed to cause MCD and FSGS [33–36]. Particularly a causative role for T-cells has long been postulated for the disease progression of MCD [32, 37–39]. In addition, the role of B-cells in the pathogenesis of MCD is under debate as depletion of B-cells via rituximab has positive effects on nephrotic syndrome including MCD [38, 40, 41] but there has also been other data that suggests B-cells do not have a causative role in MCD [42, 43].

#### 2.2.1. Guideline Recommendations for MCD Therapy

KDIGO guidelines recommend the usage of corticosteroids to induce remission in adults with MCD [1]. However, the evidence for corticosteroids comes from several large RCTs in children [44] and only small RCTs and observational studies in adults [45–48]. The recommended dosage of prednisone or prednisolone is 1 mg/kg per day (maximum 80 mg) or 2 mg/kg every other day (maximum 120 mg) for 4–16 weeks, tapered slowly over 6 months [1]. In adults, more than 50% of patients will relapse and one-third will become frequent relapsers [1]. 10–20% of the patients are steroid resistant, defined as no response to 16 weeks of oral prednisolone [32]. For patients with frequent relapses and steroid resistance, KDIGO guidelines suggest alkylating agents (oral cyclophosphamide 2–2,5 mg/kg per day for 8 weeks) [1]. In addition, if there are contraindications for alkylating agents, calcineurin inhibitors could be used [1]. As described above, only observational studies support this recommendation as of today.

#### 2.2.2. Clinical Advances and RCTs in MCD

New RCTs on nephrotic syndrome in children have been published since the publication of the KDIGO guidelines in 2012 (for review see [32, 49]). In contrast, not many new studies, especially RCTs, have been conducted in adults since 2012. In children with steroid-sensitive and steroid resistant nephrotic syndrome rituximab was effective in recently published RCTs [50, 51]. In adults, two retrospective analyses described patients with steroid-dependent or frequently relapsing MCD despite immunosuppressive therapy treated with rituximab [52, 53]. Both case series found an increase in remission in about 60% of patients. The first prospective cohort study compared rituximab treatment in 25 patients with steroid-dependent and frequently relapsing MCD to historical controls and confirmed reduction of relapses in adults with MCD [54]. A follow-up study to this prospective cohort study showed 8 relapses in 24 months after complete remission compared to 108 episodes in 24 months before rituximab [55]. A multicenter, longitudinal, intrapatient controlled trial (NEMO study) evaluated the effects of rituximab therapy followed by immunosuppression withdrawal on disease recurrence in children (*n* = 10) and adults (*n* = 20) with frequently relapsing or steroid-dependent MCD and FSGS [56]. All patients received rituximab at one (*n* = 28) or two doses (375 mg/m^2^). During the observation period (1 year), rituximab reduced relapses 5-fold compared to the time before rituximab treatment. Treatment with rituximab was safe in this patient cohort. No RCTs in adults have been conducted comparing rituximab treatment in either frequently relapsing or steroid-dependent patients or as a first-line therapy of MCD.

HP Acthar gel (ACTH), as described above, has been used in different underlying causes of nephrotic syndrome including MCD [57]. In a retrospective case study series, two patients with MCD treated with HP Acthar gel showed complete remission of the nephrotic syndrome. However, no larger trials have evaluated ACTH in MCD in adults yet.

Ongoing RCTs in adult patients with MCD investigate tacrolimus with and without low dose corticosteroids compared with high dose corticosteroids (MinTac study NCT00982072 and T-OPTIMUM trial NCT01763580). In addition, therapy with MMF plus low dose corticosteroids compared to high dose prednisolone will be investigated in a third trial (NCT01185197).

#### 2.2.3. Potential New Targets and Therapeutic Strategies in MCD

Localized upregulation and secretion of hyposialylated glycoprotein angiopoietin-like 4 (Angptl4) from podocytes is one of the key features in rodent models of MCD [58, 59]. Converting hyposialylated Angptl4 to sialylated protein using *N*-acetyl-D-mannosamine, a precursor of sialic acid that can be taken up and stored in podocytes, significantly reduces proteinuria and has the potential for use in small maintenance doses to prevent MCD relapse in rats [60].

Pioglitazone (a PPAR-*γ* agonist) has shown to slowly progress diabetic nephropathy and to reduce proteinuria. Hence, Agrawal et al. tested whether pioglitazone would enhance the efficacy of glucocorticoids in reducing proteinuria in puromycin aminoglycoside- (PAN-) induced nephrotic syndrome in rats [61]. PAN-induced nephrotic syndrome is a model for FSGS and MCD. In the study by Agrawal et al., glucocorticoids and pioglitazone not only reduced proteinuria in rats but also enhanced efficacy of glucocorticoids in reducing proteinuria by restoring podocyte marker expression, reducing Cox-2 expression, and phosphorylation of the glomerular glucocorticoid receptor [61]. Translation of these findings to a child with refractory nephrotic syndrome showed that pioglitazone reduced proteinuria by 80% and the overall immunosuppression to 64% [61]. However, in this study PAN nephrosis was induced at the same time as beginning the immunosuppressive regimen. Further studies need to investigate the role of pioglitazone as additional therapy for nephrotic syndrome.

Targeting the podocyte cytoskeleton, which is essential in maintaining the glomerular integrity, has gathered more attention lately [62]. Schiffer et al. investigated the role of endocytic protein dynamin regulating the oligomerization of the actin cytoskeleton of podocytes [63]. Targeting dynamin with Bis-T-23 reduced proteinuria in diverse proteinuric models including PAN nephrosis and diverse rodent models [63]. Targeting specifically the actin oligomerization is a promising target for diverse proteinuric kidney diseases.

### 2.3. Focal Segmental Glomerulosclerosis

Focal segmental glomerulosclerosis (FSGS) is a leading cause of ESRD in the United States and accounts for about 40% of idiopathic nephrotic syndromes in adults [64]. FSGS is diagnosed and classified from renal biopsies [65, 66]. Injury of podocytes initiates the disease process, leading to the classical focal distribution of sclerosis with a segmental pattern within the glomeruli [67]. The etiology is still unknown, but circulating permeability factors have been implicated for a long time [34–36]. As previously mentioned for MCD, patients with FSGS have high relapse rates (up to 40%) [1]. Kidney survival depends on the extent and persistence of proteinuria [1]. Patients with nonnephrotic proteinuria have a good prognosis. A significant minority will not respond to therapy; therefore the benefits and risks of the chosen immunosuppression must be weighed carefully [1].

#### 2.3.1. Guideline Recommendations for FSGS Therapy

Similar to the therapy of MCD, idiopathic FSGS should be treated with corticosteroids according to KDIGO guidelines [1]. Data to support this recommendation is based only on observational studies [1]. The duration of high dose corticosteroid therapy may be extended to 16 weeks if remission is not achieved earlier and can be tapered slowly over an additional period of 6 months [1]. Calcineurin inhibitors can be considered as first-line therapy in patients with contraindications for corticosteroid therapy [1]. Relapsing FSGS should be treated as relapsing MCD (as described above) [1]. There is no agreed definition for steroid resistance in the literature; however KDIGO guidelines suggest that corticosteroids should be given for 16 weeks before steroid resistance is diagnosed. In two RCTs, cyclosporine was tested against no therapy for steroid resistant FSGS. Remission rates were higher in the cyclosporine group [68–70]. Uncontrolled studies have found tacrolimus to be an alternative therapy to cyclosporine in primary FSGS [71, 72].

#### 2.3.2. Clinical Advances and RCTs in FSGS

Laurin et al. analyzed retrospectively a cohort of 458 patients diagnosed with primary FSGS [73]. The study found a significant association between treatment with immunosuppressive therapy (corticosteroids or CNIs) and better renal survival. There was no superiority between the two immunosuppressive modalities used in this study.

A small, single-center study of adults with idiopathic FSGS compared intravenous monthly cyclophosphamide plus steroids to tacrolimus plus steroids for 6 months [74]. Both groups had improved proteinuria and serum albumin with stable GFR but the results were statistically not significant. In an uncontrolled trial of 44 adults with steroid resistant FSGS treated with tacrolimus for 24 weeks, half of the patients achieved complete or partial remission [75].

Case reports and small case series have shown potential for rituximab in steroid-sensitive FSGS, but it appears largely ineffective in steroid resistant disease [76, 77].

A recent small series (*n* = 15) examined adrenocorticotropic hormone gel (HP Acthar gel) in idiopathic FSGS. Sixty percent of patients showed partial remission [57].

Several case reports have shown conflicting results on the treatment of FSGS patients with galactose, which was found to bind potential circulating permeability factors in FSGS [78–80]. Recently, the FONT II trial was published as a phase I/II open label randomized controlled trial comparing standard conservative therapy (SCT) versus SCT plus adalimumab (antibody against tumor necrosis factor-*α*/TNF-*α*) versus SCT plus galactose [81]. The primary end point was a 50% reduction of proteinuria with stable GFR. Seven out of 21 patients received SCT plus galactose and three of them met the primary end point. No improvement was noted in the SCT plus adalimumab group. Further studies are needed to evaluate the role of galactose in the treatment of FSGS.

Abatacept, a costimulatory inhibitor of B7-1 (CD80), induced remission in five patients with idiopathic or recurrent FSGS [82]. One case report confirmed the positive effect of abatacept in a patient with FSGS recurrence in the transplanted organ [83]. However, others could not confirm the overexpression of B7-1 or the effects of abatacept in similar patients [84–87].

Ofatumumab is a fully human monoclonal antibody against CD20. It has recently been shown to be effective in rituximab-resistant nephrotic patients in case reports and small case series (mostly children) with nephrotic syndrome [32, 41, 88, 89]. In children, two RCTs are recruiting patients to test ofatumumab versus placebo in drug resistant nephrotic syndrome (clinicaltrials.gov NCT02394106) and ofatumumab versus rituximab in drug resistant nephrotic syndrome (clinicaltrials.gov NCT02394119). So far, there are no planned or published RCTs or case reports investigating ofatumumab in adults with FSGS.

As described above, HP Acthar gel is currently evaluated in an interventional study for patients with FSGS undergoing renal transplantation (clinicaltrials.gov NCT02683889). In addition, tacrolimus therapy is currently tested in a randomized study compared to therapy with cyclophosphamide in patients with FSGS (clinicaltrials.gov NCT01451489).

Fresolimumab, a human monoclonal antibody neutralizing TGF-beta, was tested in a phase 2 RCT in steroid resistant patients with primary FSGS (clinicaltrials.gov NCT01665391) [90]. Even though the primary and secondary endpoints were not achieved as the study was underpowered, the AE profile was safe [90]. Further studies need to evaluate the role for Fresolimumab in therapy of steroid resistant primary FSGS.

#### 2.3.3. Potential New Targets and Therapeutic Strategies in FSGS

Delville et al. described the identification of an autoantibody panel in recurrent FSGS before transplantation [91]. CD40 is expressed in human cultured podocytes and its expression cannot be induced by challenging in vitro [91]. However, in patients with FSGS, CD40 was detected in glomeruli from recurrent FSGS patients [91]. Purified CD40 autoantibodies from recurrent FSGS sera disrupted the podocyte (human) actin cytoskeleton in vitro [91]. These data suggested that suPAR-*β*_3_-integrin pathway could be involved [91]. Building on these exciting findings, the role of CD40 antibodies in human FSGS disease needs to be further validated. Anti-CD40 blocking antibodies (ASKP1240 or lucatumumab) are already commercially available and could become potential treatment options tested in clinical trials [36].

Urokinase plasminogen activator receptor (uPAR) is a cell membrane glycosylphosphatidylinositol- (GPI-) anchored protein expressed in many cell types, for example, immune cells [92–94], endothelial cells [95], tumor cells [96], tubular epithelial cells [97], and podocytes [98]. Through cleavage of uPAR from its GPI-anchor, the soluble urokinase plasminogen activator receptor (suPAR) is released. Recently, suPAR has emerged as a biomarker in different disease conditions. For example, suPAR concentrations were associated with increased risk of cardiovascular events in the general population [99, 100]. Several studies have described an inverse correlation of suPAR levels with the estimated glomerular filtration rate (eGFR) [101–104]. Since its first description as a permeability factor in FSGS by Wei et al. [105], many researchers have published conflicting results concerning the causative role of suPAR for FSGS [101, 106]. Amiloride, a potassium sparing diuretic, has recently been shown to reduce uPAR expression in podocytes in vitro and in proteinuric rodent models in vivo [107]. Amiloride has been used in case reports for patients with Alport and Fabry's disease to reduce proteinuria in addition to standard therapy [108, 109]. There have been no reports on amiloride therapy in humans with FSGS.

Saquinavir, an HIV protease inhibitor with proteasome inhibiting activity, has been tested in a pilot study and in case reports in patients with a long history of nephrotic syndrome despite immunosuppressive therapy [110, 111]. In this study, saquinavir was used as an add-on therapy to other immunosuppressive agents. One out of four primary-steroid resistant nephrotic syndrome patients and five out of six steroid-dependent nephrotic syndrome patients became infrequent (5) or frequent (1) relapsers [110]. Immunosuppressive dosages, especially corticosteroids, were significantly reduced [110]. In podocytes and peripheral blood mononuclear monocytes, saquinavir blunted NF-*κ*B activation [110]. The observation that the proteasome inhibitor was beneficial in FSGS warrants further investigation and larger studies.

As described above the podocyte cytoskeleton is also an interesting new target in FSGS (see section potential new targets and therapeutic strategies in MCD).

## 3. Conclusion

Major advances in the pathophysiological understanding, especially of iMN, have led to new treatment strategies in the past years. However, even though the incidence of primary causes of nephrotic syndrome is low, the lack of larger RCTs in this field is striking [112]. Therefore, adult patients with nephrotic syndrome should be treated within clinical trials in the future. Many interesting new treatment targets identified by basic science have been proposed. Further investigation of these new targets and those identified in the future will potentially lead to novel advances in the treatment of nephrotic syndrome, with higher effectiveness in reducing proteinuria at an acceptable level of AEs.
